# Levosimendan Pretreatment Attenuates Mesenteric Artery Ischemia/Reperfusion Injury and Multi-Organ Damage in Rats

**DOI:** 10.3390/ijms26189131

**Published:** 2025-09-18

**Authors:** Zoran Matković, Milica Gajić Bojić, Uglješa Maličević, Aleksandra Krivokuća, Nebojša Mandić-Kovačević, Snežana Uletilović, Ljiljana Amidžić, Sanja Jovičić, Maja Barudžija, Miloš P. Stojiljković, Radoslav Gajanin, Sergej Bolevich, Ranko Škrbić

**Affiliations:** 1 Department of Surgery, General Hospital “Sveti Apostol Luka”, 74000 Doboj, Bosnia and Herzegovina; zoranmatna@gmail.com; 2 Centre for Biomedical Research, Faculty of Medicine, University of Banja Luka, 78000 Banja Luka, Bosnia and Herzegovina; milica.gajic@med.unibl.org (M.G.B.); ugljesa.malicevic@med.unibl.org (U.M.); aleksandra.krivokuca@med.unibl.org (A.K.); nebojsa.mandic-kovacevic@med.unibl.org (N.M.-K.); snezana.uletilovic@med.unibl.org (S.U.); ljiljana.amidzic@med.unibl.org (L.A.); sanja.jovicic@med.unibl.org (S.J.); maja.barudzija@med.unibl.org (M.B.); milos.stojiljkovic@med.unibl.org (M.P.S.); radoslav.gajanin@med.unibl.org (R.G.); 3 Department of Pharmacology, Toxicology and Clinical Pharmacology, Faculty of Medicine, University of Banja Luka, 78000 Banja Luka, Bosnia and Herzegovina; 4 Department of Pathophysiology, Faculty of Medicine, University of Banja Luka, 78000 Banja Luka, Bosnia and Herzegovina; 5 Department of Pharmacy, Faculty of Medicine, University of Banja Luka, 78000 Banja Luka, Bosnia and Herzegovina; 6 Department of Medical Biochemistry and Chemistry, Faculty of Medicine, University of Banja Luka, 78000 Banja Luka, Bosnia and Herzegovina; 7 Department of Human Genetics, Faculty of Medicine, University of Banja Luka, 78000 Banja Luka, Bosnia and Herzegovina; 8 Department of Histology and Embryology, Faculty of Medicine, University of Banja Luka, 78000 Banja Luka, Bosnia and Herzegovina; 9 Department of Pathology, University Clinical Centre of the Republic of Srpska, 78000 Banja Luka, Bosnia and Herzegovina; 10 Academy of Sciences and Arts of the Republic of Srpska, 78000 Banja Luka, Bosnia and Herzegovina; 11 Department of Pathologic Physiology, First Moscow State Medical University “I.M. Sechenov”, 119435 Moscow, Russia; bolevich2011@yandex.ru

**Keywords:** ischemia/reperfusion, mesenteric artery, levosimendan, oxidative stress, inflammation, apoptosis

## Abstract

Acute mesenteric ischemia (AMI) is a life-threatening condition characterised by oxidative stress, inflammation, apoptosis, and necrosis of intestinal epithelial cells. Different drugs with vasoactive, antioxidant, and anti-inflammatory properties have been used to treat AMI. Levosimendan is a drug with proven anti-ischemic effects used in the management of acute congestive heart failure. This study evaluated the protective effects of levosimendan pretreatment on intestinal, as well as lung, heart, and kidney tissue in a rat model of mesenteric artery ischemia/reperfusion (I/R) injury. Male Wistar rats (N = 24) were divided into four groups: control, I/R, levosimendan (LS) 1 mg/kg i.p, and LS + I/R (1 mg/kg i.p. 30 min before injury). I/R by itself caused elevation of oxidative markers (thyobarbituric acid reactive species (TBARS), hydrogen peroxide (H_2_O_2_), super oxide anjon radical (O_2_^−^), and nitrogen dioxide (NO_2_^−^)), induced inflammation (macrophage infiltration and Interleukin-6 (IL-6) production), and apoptosis (nuclear factor kappa light-chain enhancer of activated B cells (NF-κB), cleaved caspase-3 (CC3), and terminal deoxy-nucleotidyl transferase (TdT)-mediated dUTP nick end labelling (TUNEL)). Levosimendan pretreatment significantly reduced oxidative stress markers and enhanced antioxidant defences (catalase (CAT), reduced glutathione (GSH), and superoxide dismutase (SOD)). Histological analysis revealed reduced mucosal damage and preserved goblet cells in intestinal tissue. Similar protective effects of levosimendan were observed in other organs such as lung, heart, and kidney. Immunohistochemistry showed reduced epithelial apoptosis and upregulation of antioxidant and anti-inflammatory proteins. These findings highlight levosimendan’s ability to protect mesenteric I/R tissue injury and multi-organ damage by suppressing oxidative stress, inflammation, and apoptosis, emphasising its therapeutic potential in clinical settings.

## 1. Introduction

AMIis defined as insufficient perfusion in the mesenteric vascular bed caused by inadequacy of the arterial supply or venous drainage of the intestine [1]. The interrupted blood flow is followed by intestinal ischemia and tissue oedema with further progression of intestinal gangrene, manifested as peritonitis, multi-organ dysfunction syndrome (MODS), cardiovascular collapse, and death. Although its incidence is low (6.2 per 100,000 inhabitants), AMI is a potentially fatal vascular, gastrointestinal, and surgical condition with a high mortality rate of 60–80% [2,3]. Despite the progress in the understanding of pathogenesis and development of modern treatment modalities, AMI remains a clinical challenge [4,5]. The current treatment options, like the early administration of anticoagulants, vasodilators, and thrombolytic therapy, are primarily oriented toward revascularisation and restoration of mesenteric blood flow [6]. It was found that the restoration of circulation and the release of reactive oxygen species (ROS) cause even greater damage to the intestine than the previous ischemia itself [2,7]. While mesenteric ischemia disturbs the function of intestinal villi, reperfusion following blood flow restoration exacerbates ischemic damage that would induce intestinal necrosis. This can raise the risk of bacterial translocation and sepsis, as well as the risk of MODS, which is the main cause of death in patients with mesenteric I/R injury [8].

Intestinal (I/R) injury involves various cascades like production of ROS, mitochondrial alteration, intestinal permeability disturbance, activation of innate immune response, and production of pro-inflammatory mediators leading to induction of apoptotic signalling pathways [8]. The activation of innate immune response following mesenteric I/R enhance toll-like receptor 4 (TLR4)/ NF-κB pathway and subsequently alleviate both systemic and intestinal levels of tumour necrosis factor alpha (TNF-α) and IL-6 [9,10].

According to therapeutic guidelines, the treatment of AMI includes different conservative procedures, such as intravenous rehydration, anticoagulant, analgetic, thrombolytic, and antiplatelet therapy. These could be combined with additional surgical protocols, including endovascular arterial revascularisation or bowel resection [6,11].

Different procedures and drugs have been used to attenuate AMI. Drugs with vasoactive, antioxidant, and anti-inflammatory effects have been investigated in experimental models of AMI [12]. It has been shown that milrinone and levosimendan can improve the perfusion of the primary area of ischemia, without effect on the splanchnic vasoconstriction [12,13]. Levosimendan is a drug with proven anti-ischemic effects, attributed to numerous pleiotropic effects, including antioxidant, anti-inflammatory, and anti-apoptotic effects. As a calcium sensitiser in the cell, levosimendan leads to greater cardiac muscle fibre strength without increasing energy expenditure, as reflected by its anti-ischemic activity. Additionally, levosimendan is a powerful vasodilator through the opening of adenosine triphosphate (ATP) -dependent potassium channels in the smooth muscle cells [13]. Apart from the myocardium, levosimendan causes vasodilation in other organs, including the lungs, intestines, liver, and kidneys. Its active metabolite, OR 1896, with a very long half-life of 80 h, is responsible for the prolonged beneficial effect [14]. However, levosimendan is not used in the treatment of patients with AMI, and it has been rarely used even in preclinical studies with experimental AMI models [15,16].

The aim of this study was to investigate the protective effects of levosimendan on intestinal mucosa and multi-organ damage, following mesenteric I/R injury in rats through evaluation of oxidative stress, tissue inflammation, and apoptosis.

## 2. Results

### 2.1. Levosimendan Attenuates the Effects of Mesenteric I/R Injury on Oxidative Stress Markers in Blood, Intestinal Tissue, and Bronchoalveolar Lavage Fluid

**Figure 1:**
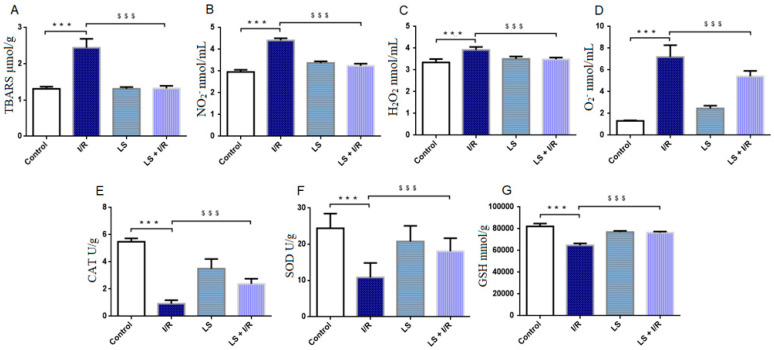
Effects of levosimendan (LS) on oxidative stress markers in rat plasma and erythrocyte lysate. (**A**) TBARS (μmol/g); (**B**) NO_2_^−^ (nmol/mL); (**C**) H_2_O_2_ (nmol/mL); (**D**) O_2_^−^ (nmol/mL); (**E**) CAT (U/g); (**F**) SOD (U/g); (**G**) GSH (mmol/g). Mesenteric I/R injury led to a significant increase in the lipid peroxidation index (TBARS) and pro-oxidative markers: H_2_O_2_, NO_2_^−^, and O_2_^−^, along with a decrease in antioxidant enzymes (CAT, SOD, and GSH), while pretreatment with levosimendan significantly reduced plasma TBARS and pro-oxidative markers and enhanced antioxidant enzyme levels compared to the I/R group. Data are expressed as mean ± SD. Control (N = 6), I/R (N = 6), LS (N = 6), LS + I/R (N = 6). *** *p* < 0.001 comparing control with I/R group; ^$$$^ *p* < 0.001 comparing I/R with LS + I/R group.

A significant increase in the lipid peroxidation index (TBARS) was observed in I/R group compared to the control. This notable increase in plasma TBARS was associated with elevated levels of all the tested pro-oxidative markers, like H_2_O_2_, O_2_^−^, and NO_2_ (*p* < 0.05). The I/R group also showed a decrease in levels of antioxidative enzymes CAT, SOD, and GSH, as measured in erythrocyte lysate. However, pretreatment with levosimendan mitigated the effects of mesenteric I/R injury, as evidenced by a significant reduction in plasma levels of TBARS and pro-oxidative enzymes (*p* < 0.05). Additionally, pretreatment with levosimendan exhibited an antioxidant effect by increasing the levels of antioxidant enzymes, SOD, CAT, and GSH, compared to I/R group (Figure 1).

**Figure 2:**
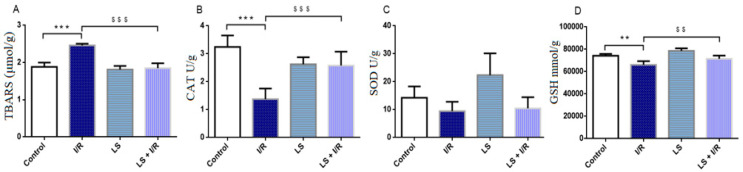
Effects of levosimendan (LS) on oxidative stress markers in rat intestinal tissue homogenate. (**A**) TBARS (μmol/g); (**B**) CAT (U/g); (**C**) SOD (U/g); (**D**) GSH (mmol/g). TBARS levels are significantly increased and CAT, SOD, and GSH activities are significantly decreased in I/R groups compared to controls, while levosimendan pretreatment significantly reduced TBARS levels and improved CAT and GSH activities, but had no beneficial effect on SOD activity. Data are expressed as mean ± SD. Control (N = 6), I/R (N = 6), LS (N = 6), LS + I/R (N = 6). ** *p* < 0.01, *** *p* < 0.001, comparing control with I/R group; ^$$^ *p* < 0.01, ^$$$^ *p* < 0.001, comparing I/R with LS + I/R group.

Consistent with plasma findings, TBARS levels were significantly (*p* < 0.05) elevated in terminal ileum tissue homogenates of rats in the I/R group compared to the control group. In addition, CAT, SOD, and GSH activities were significantly decreased in the terminal ileum tissue homogenates of rats subjected to I/R compared to controls (*p* < 0.05). The elevated values of TBARS were restored and antioxidative CAT and GSH activities were significantly improved in I/R group pretreated with levosimendan (*p* < 0.05). However, levosimendan pretreatment did not demonstrate beneficial effects on SOD activity in ileum tissue homogenate (Figure 2).

**Figure 3:**
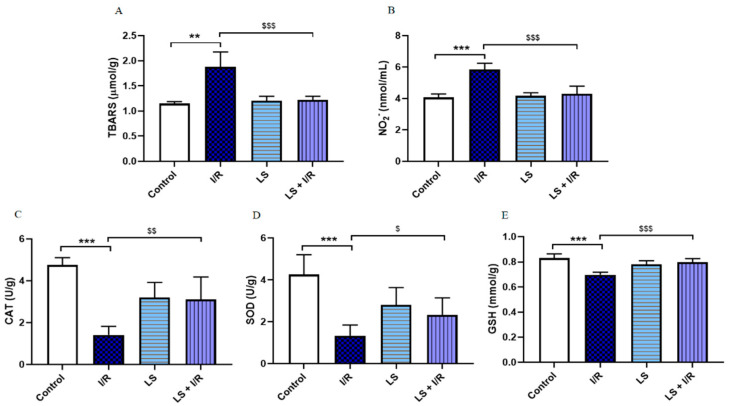
Effects of levosimendan (LS) on oxidative stress markers in rat BALF. (**A**) TBARS (μmol/g); (**B**) NO_2_^−^ (nmol/mL); (**C**) CAT (U/g); (**D**) SOD (U/g); (**E**) GSH (mmol/g). TBARS levels are significantly increased and CAT, SOD, and GSH activities are significantly decreased in I/R groups compared to controls, while levosimendan pretreatment significantly reduced TBARS levels and improved CAT, SOD, and GSH activities. Data are expressed as mean ± SD. Control (N = 6), I/R (N = 6), LS (N = 6), LS + I/R (N = 6). ** *p* < 0.01; *** *p* < 0.001 comparing control with I/R group; ^$^ *p* < 0.05; ^$$^ *p* < 0.01; ^$$$^ *p* < 0.001, comparing I/R with LS + I/R group.

Levosimendan modulated oxidative stress markers in rat bronchoalveolar lavage fluid (BALF). In the I/R group, TBARS (*p* < 0.01) and NO_2_^−^ (*p* < 0.001) levels were markedly elevated, whereas CAT, SOD, and GSH activities were significantly reduced compared to controls (*p* < 0.001). Pretreatment with levosimendan attenuated the increase in TBARS and NO_2_^−^ (*p* < 0.001) and significantly restored CAT (*p* < 0.01), SOD (*p* < 0.05), and GSH (*p* < 0.001) activities toward control levels (Figure 3).

### 2.2. Protective Effects of Levosimendan on Rat Terminal Ileum Epithelial Cells Induced by I/R Injury

**Figure 4:**
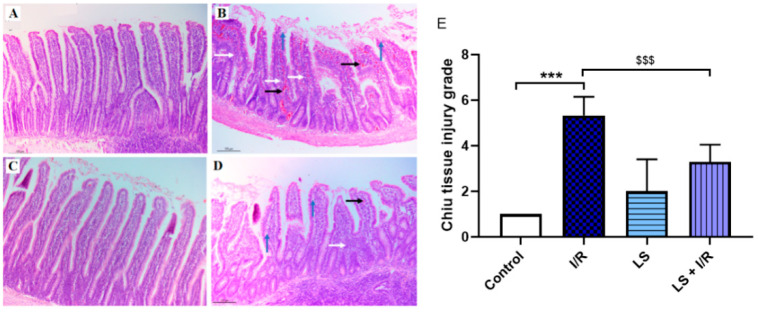
Representative microphotographs of rat terminal ileum sections stained by haematoxylin and eosin (magnification 10×, scale bar = 100 µm). (**A**) Preserved architecture of the terminal ileum mucosal villi in the control group. (**B**) The I/R group displays severe disruption of mucosal villi architecture with extensive epithelial lifting (blue arrows), inflammatory cell infiltration (white arrows), and haemorrhage (black arrows) within the lamina propria. (**C**) LS group showed normal terminal ileum histology. (**D**) The LS + I/R group exhibits notably reduced mucosal damage, with milder inflammatory infiltration and haemorrhage compared to the I/R group. (**E**) Terminal ileum injury was graded according to the Chiu scoring system (mean ± SD, Control (N = 6); I/R (N = 6); LS (N = 6); LS + I/R (N = 6); 10 fields per sample). *** *p* < 0.001; ^$$$^ *p* < 0.001 (between indicated groups).

As shown in Figure 3, the ileum sections of the I/R group displayed disruption of the mucosal villi architecture, associated with significant epithelial lifting, inflammatory infiltration, and haemorrhage. The LS group displayed histological findings comparable to the control. Pretreatment with levosimendan showed significantly less disintegration of the mucosal villi, along with reduced inflammatory infiltration and haemorrhage in the lamina propria, compared to the I/R group. The terminal ileum injury grade, based on the Chiu score, was used to semi-quantify the differences between groups stained by haematoxylin and eosin (Figure 4).

**Figure 5:**
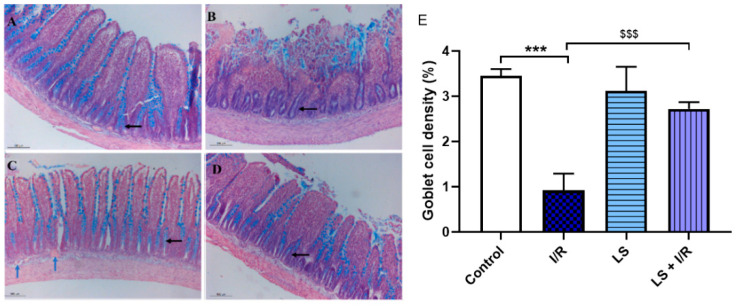
Effects of levosimendan (LS) on histopathology of intestinal tissue in mesenteric I/R injury. Small intestine cross section, magnification ×100 (Alciane blue stain, scale bar 100 µm). (**A**) Control group shows normal terminal ileum histology, with a typical distribution of goblet cells in Lieberkühn’s crypts (black arrow) and epithelial layer of the mucosa. (**B**) I/R group shows destruction of the apical part of villi, inflammatory infiltrate at the base, and absence of goblet cells in crypts (black arrow). (**C**) LS group exhibits a thickened submucosa with dilated blood vessels (blue arrows) and normal mucosa histology with goblet cells in crypts (black arrow). (**D**) LS + I/R group shows dilated villi, mostly normal goblet cell distribution, but occasional absence in single crypts (black arrow). (**E**) Numerical areal density of goblet cells (%), showing significant preservation in the LS + I/R group (mean ± SD, Control (N = 6), I/R (N = 6), LS (N = 6), LS + I/R (N = 6); 10 fields per sample). *** *p* < 0.001; ^$$$^ *p* < 0.001 (between indicated groups).

The histopathologic examination of the rat terminal ileum stained with Alcian blue revealed findings similar to those previously observed with haematoxylin and eosin staining. The control group shows normal terminal ileum histology with a typical distribution of goblet cells in the crypts of Lieberkühn and the epithelial layer of the mucosa. The I/R group showed destruction of villi, inflammatory infiltrate, and absence of goblet cells in the crypts. In the LS group a normal distribution of goblet cells was seen, while the pretreatment with levosimendan in I/R group had a mostly normal distribution of goblet cells, with occasional absence in some crypts (Figure 5).

### 2.3. Effects of Levosimendan on Inflammatory Response of Intestinal Tissue Induced by Mesenteric I/R Injury

**Figure 6:**
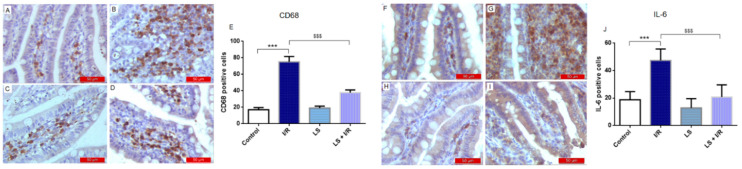
Immunohistochemical results of the expression of CD68 (left panel) and IL-6 (right panel) in rat small intestinal tissue. Data are expressed as mean ± SD. Representative immunohistochemical images at 400× magnification. (**A**,**F**) Control groups. (**B**,**G**) A marked elevation in the number of CD68-positive macrophages (**B**) and IL-6-positive cells (**G**) was observed in the I/R groups. (**C**,**H**) LS group exhibited similar immunoreactivity to the control. (**D**,**I**) The LS + I/R group exhibited significantly decreased immunoreactivity for both macrophages and IL-6. (**E**,**J**) Percentage of diaminobenzidin (DAB) -stained cells in small intestine tissue indicating the proportion of CD68-positive (**E**) and IL-6-positive (**J**) cells. *** *p* < 0.001 comparing control with I/R group; ^$$$^ *p* < 0.001 comparing I/R with LS + I/R group.

In addition to causing structural damage and depletion of antioxidant defences, mesenteric I/R injury resulted in a pronounced elevation of inflammatory mediators. CD68 and IL-6 immunoreactivity indicated their key involvement in initiating and sustaining inflammation in I/R injury. In the control group, only a small number of CD68-positive macrophages and IL-6-positive cells were observed in the stromal region outside the blood vessels within the villi. In contrast, the I/R group showed a significant increase in CD68-positive macrophages and IL-6-positive cells, predominantly localised at the erosion fronts of the damaged villi. The levosimendan group showed immunoreactivity comparable to the control group, whereas levosimendan pretreatment significantly reduced macrophage infiltration and IL-6 expression in I/R-injured tissue (Figure 6).

### 2.4. Levosimendan Attenuated Apoptosis of Intestinal Epithelial Cells Induced by I/R Injury

**Figure 7:**
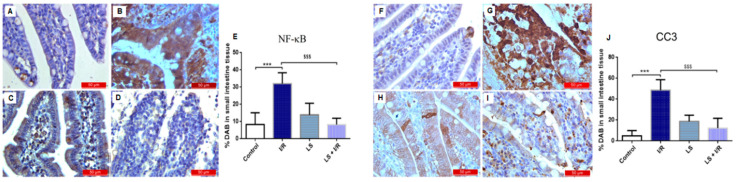
Immunohistochemical results of the expression of NF-kB (left panel) and CC3 (right panel) in rat small intestinal tissue. Data are expressed as mean ± SD. Representative immunohistochemical images at 400× magnification. (**A**,**F**) Control groups. (**B**,**G**) Intense cytoplasmic staining of NF-κB (**B**) and CC3 (**G**) in intestinal epithelial cells of the I/R groups, indicative of apoptosis-related activation. (**C**,**H**) LS groups showed no significant difference in immunoreactivity to apoptosis markers compared to the control groups. (**D**,**I**) Marked reduction in epithelial apoptosis and intestinal damage in the LS + I/R groups. (**E**,**J**) Percentage of DAB-stained cells in small intestine tissue indicating the proportion of NF-κB-positive (**E**) and CC3-positive (**J**) cells. *** *p* < 0.001 comparing control with I/R group; ^$$$^ *p* < 0.001 comparing I/R with LS + I/R group.

Immunohistochemistry analysis revealed a significant upregulation of NF-κB and CC3. NF-kB and CC3 in the I/R group, indicating that these markers play a key role in the pathophysiological response to rat ileum damage and apoptosis. Pretreatment with levosimendan (1 mg/kg) significantly attenuated intestinal epithelial cell apoptosis induced by I/R injury, as evidenced by downregulation of NF-kB and CC3 expression. The results showed that levosimendan pretreatment could alleviate I/R-induced intestinal injury (LS + I/R vs. I/R, *p* < 0.001) (Figure 7).

**Figure 8:**
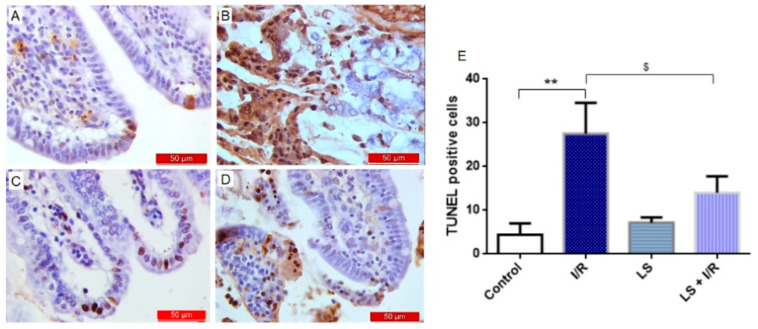
Levosimendan inhibited apoptosis in rat small intestinal tissue in mesenteric I/R injury detected by TUNEL staining, magnification 400×. (**A**) Control group. (**B**) A significant increase in DNA fragmentation was observed in the I/R group, evidenced by intensified brown TUNEL staining. (**C**) The LS group exhibited a similar appearance and distribution of TUNEL-positive cells as the control group. (**D**) Pretreatment with levosimendan prevented I/R-induced nuclear apoptosis. (**E**) Quantitative analysis of apoptotic cells was performed on immunohistochemically stained sections of rat intestinal tissue. ** *p* < 0.01 comparing control with I/R group; ^$^ *p* < 0.05 comparing I/R with LS + I/R group.

To further confirm the anti-apoptotic effects of LS, a TUNEL analysis (terminal deoxy-nucleotidyl transferase (TdT)-mediated dUTP nick end labelling) was performed. Levosimendan pretreatment decreased apoptotic index, which was observed as a significantly decreased number of mostly TUNEL-positive epithelial cells of intestinal tissue (Figure 8).

### 2.5. Levosimendan Pretreatment Induced Upregulation of Nrf2, HO-1, and Nrf2/HO-1 Signalling

**Figure 9:**
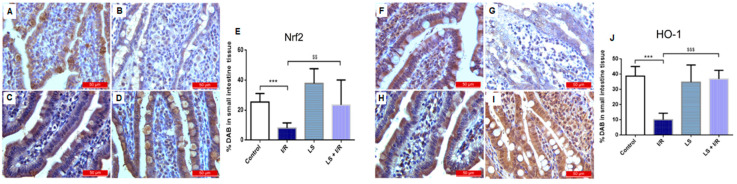
Immunohistochemical results of the expression of Nrf2 (left panel) and HO-1 (right panel), in rat small intestinal tissue. Data are expressed as mean ± SD. Representative immunohistochemical images at 400× magnification. (**A**,**F**) Control groups. (**B**,**G**) Marked reduction in immunoreactivity for the antioxidant markers Nrf2 (**B**) and HO-1 (**G**) was observed in intestinal epithelial cells of the I/R group. (**C**,**H**) Immunoreactivity for both markers in the LS group was comparable to that observed in the control group, with no significant differences detected. (**D**,**I**) Pretreatment with levosimendan significantly increased antioxidant marker levels compared with the I/R group. (**E**,**J**) Percentage of DAB-stained cells in small intestine tissue indicating the proportion of Nrf2-positive (**E**) and HO-1-positive. (**J**) cells. *** *p* < 0.001 comparing control with I/R group; ^$$^ *p* < 0.01; ^$$$^ *p* < 0.001 comparing I/R with LS + I/R group.

Furthermore, the results indicate that overexpression of nuclear factor erythroid 2- related factor (Nrf2), as well as the hem oxygenase 1 (HO-1) antioxidant response in the terminal ileum reduce intestinal I/R injury. The control and levosimendan pretreated groups showed similar appearance and distribution of Nrf2- and HO-1-positive cells, with no significant differences observed, whereas the I/R group exhibited a marked decrease in immunoreactivity. Levosimendan successfully elevated the levels of these antioxidant markers, as evidenced by significant differences between the LS + I/R and I/R groups (Nrf2, *p* < 0.01; HO-1, *p* < 0.001) (Figure 9).

### 2.6. Protective Effects of Levosimendan on Heart, Lung, and Kidney Induced by Mesenteric Artery I/R Injury

**Figure 10:**
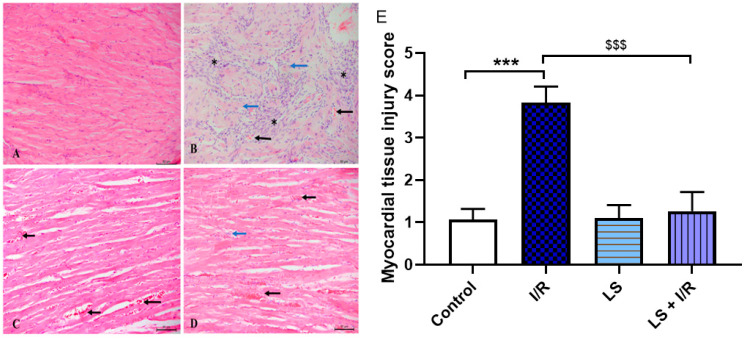
Representative microphotographs of rat cardiac muscle longitudinal sections stained with haematoxylin and eosin (magnification ×20, scale bar = 50 µm). (**A**) Preserved histological structure of the cardiac muscle in the control group. (**B**) The I/R group displays dense inflammatory infiltrate permeating the cardiac muscle (asterisk); presence of damaged cardiomyocytes (blue arrow) and erythrocyte extravasation (black arrow). (**C**) LS group preserved histological structure of the cardiac muscle, with dilated blood vessels in the endomysium (black arrow). (**D**) The LS + I/R group preserved histological structure of the cardiac muscle; in addition to dilated blood vessels in the endomysium (black arrow), scattered inflammatory cells are also present (blue arrow). (**E**) Tissue damage score of cardiac muscle (mean ± SD, Control, N = 6; LS, N = 6; I/R, N = 6; LS + I/R, N = 6). *** *p* < 0.001; ^$$$^ *p* < 0.001 (between indicated groups).

I/R injury induces damage not only in thegastrointestinal tract but also in remote organs such as the heart, lungs, and kidneys. The myocardial injury is most pronounced in the septal region and apex of the heart. Histopathological examination reveals cardiomyocyte hypertrophy, extensive inflammatory infiltrates predominantly composed of lymphocytes, and consequent myocardial necrosis. Levosimendan administration demonstrates significant vasodilatory properties, as observed by the presence of dilated blood vessels engorged with erythrocytes within the endomysium. Pretreatment with levosimendan confers marked cardioprotection, reflected by improved tissue damage scores. Cardiomyocyte integrity is preserved, vascular dilation in the endomysium persists, and only sparse inflammatory cells remain evident (Figure 10).

**Figure 11:**
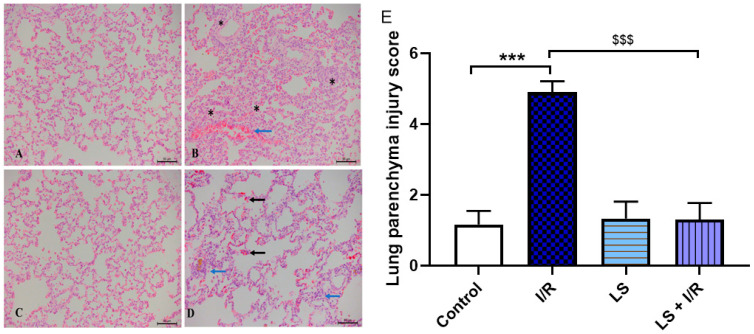
Representative microphotographs of rat lung parenchyma sections stained with haematoxylin and eosin (magnification ×20, scale bar = 50 µm). (**A**) Preserved histological structure of the lung parenchyma in the control group. (**B**) The I/R group displays severe damage, with interalveolar spaces completely filled with inflammatory infiltrate (asterisk), dilated blood vessels are present, and erythrocyte extravasation (black arrow). (**C**) LS group preserved histological structure of the lung parenchyma. (**D**) The LS + I/R group mostly preserved histological structure of the lung parenchyma; scattered inflammatory cells (blue arrow) and erythrocyte extravasation (black arrow) are present. (**E**) Tissue damage score of lung parenchyma (mean ± SD, Control (N = 6), I/R (N = 6), LS (N = 6), LS + I/R (N = 6); 10 fields per sample). *** *p* < 0.001; ^$$$^ *p* < 0.001 (between indicated groups).

The lungs are among the organs most severely affected by I/R injury. This condition leads to diffuse damage of the pulmonary parenchyma, accompanied by significant impairment of the respiratory epithelium. Nearly all alveoli exhibit structural damage and are infiltrated with dense inflammatory cells. Additionally, fibrotic changes are evident in the basal regions of the lungs. Pretreatment with levosimendan confers substantial protection to the pulmonary parenchyma, with only sparse inflammatory cell foci persisting in the basal lung areas, while the majority of the pulmonary tissue remains preserved (Figure 11).

**Figure 12:**
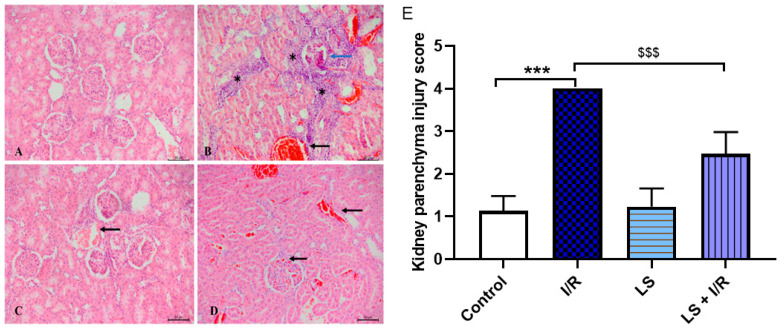
Representative microphotographs of rat renal cortex sections stained with haematoxylin and eosin (magnification ×20, scale bar = 50 µm). (**A**) Preserved histological structure of the renal cortex in the control group. (**B**) The I/R group displays dense inflammatory infiltrate (asterisk) in renal parenchyma; completely damaged renal corpuscle (blue arrow), dilated blood vessels with erythrocyte extravasation (black arrow). (**C**) LS group preserved histological structure of the renal cortex, slightly dilated blood vessel (black arrow). (**D**) The LS + I/R group preserved histological structure of the renal cortex; presence of dilated blood vessels, including glomerulus blood vessels (black arrow). (**E**) Tissue damage score of renal parenchyma (mean ± SD, Control (N = 4), I/R (N = 6), LS (N = 5), LS + I/R (N = 6); 10 fields per sample). *** *p* < 0.001; ^$$$^ *p* < 0.001 (between indicated groups).

The renal parenchyma, including both the cortex and medulla, is also susceptible to damage during mesenteric I/R injury. Dense inflammatory infiltrates permeate the cortical region, causing complete degeneration of the glomeruli. Although pretreatment with levosimendan demonstrates a protective effect on the renal parenchyma, tissue damage score analysis reveals that its protective impact is relatively limited in this tissue compared to other organs. Histological examination shows preservation of the glomeruli; however, epithelial damage persists in the collecting ducts within the medullary region, accompanied by sparse foci of inflammatory cells (Figure 12).

## 3. Discussion

In this study, we found that levosimendan pretreatment exhibits antioxidant, anti-inflammatory, and anti-apoptotic properties that are responsible for multi-organ tissue protective effects in an experimental model of mesenteric artery I/R injury in rats.

Intestinal mucosa tissue is very sensitive to hypoxia and a high amount of oxygen is required to maintain the functional integrity of the digestive tract. When AMI occurred, the oxygen deprivation initiated the intracellular blockade of mitochondrial oxidative phosphorylation, with consecutive ATP depletion and inhibition of ATPase-dependent ionic pumps. This inevitably induces the increase in intracellular sodium and calcium levels and disintegration of cytoskeleton and cellular membranes, which can finally lead to cell rupture and necrosis, particularly if ischemia is prolonged. During ischemia, ATP is gradually converted into adenosine that can be further degraded to inosine and hypoxanthine, which accumulates in the ischemic tissue [8,17].

After the tissue is reperfused, the enzyme xanthine oxidase utilises the freely available oxygen to oxidise hypoxanthine. This process is associated with O_2_^−^ production, which is then transformed by SOD into H_2_O_2_, and this molecule is further cleaved into highly reactive and cytotoxic hydroxyl radicals (·OH). Additionally, O_2_^−^ can combine with nitric oxide (NO) to produce peroxynitrite (ONOO^−^). The excessive production of reactive oxygen/nitrogen species (ROS/RNS) initiate the fragmentation of proteins and nucleic acids, as well as the lipid peroxidation of cellular membranes. All these events ultimately lead to epithelial cell apoptosis and necrosis [8]. However, a large amount of free oxygen radicals generated during the reperfusion phase cannot be removed by the endogenous antioxidant system (SOD, CAT, and GSH). Therefore, many antioxidant compounds have been investigated for reduction in the damage caused by I/R injury [18,19].

In our study, levosimendan significantly reduced the oxidative stress markers (TBARS, H_2_O_2_, and NO_2_) and increased the activity of antioxidative enzymes (CAT and SOD) and GSH, demonstrating a strong antioxidant effect in serum, intestine homogenate, and BALF. These results are in accordance with previous study, in which levosimendan showed strong antioxidative properties in carrageenan-induced inflammatory paw oedema of rat [18]. Similarly, levosimendan also alleviated the sepsis-induced cardiac dysfunction by suppressing oxidative stress and inflammation and regulating cardiac mitophagy [20], as well as the hypoxia-induced brain injury in rats by ameliorating the oxidative stress and inflammation [21].

It is known that ROS activate NF-kB and trigger the transcription of many pro-inflammatory mediators like TNF-alfa, IL-6, and IL-1. These cytokines contribute to the inflammatory response in the intestinal mucosa, where neutrophils and macrophages are a major source of these cytokines. NF-kB is linked to homeostasis and alters the permeability of the intestinal layer and intensifies the intestinal damage [22]. The strong anti-inflammatory property of levosimendan was confirmed in our study, since the pretreatment with this drug significantly reduced macrophage infiltration, IL-6, and NF-kB expression in rat intestinal tissue following I/R injury.

Intestinal wall damage is a well-recognised consequence of AMI, with the intestinal villi being highly susceptible to ischemia and epithelial necrosis representing one of the earliest histological changes [10,11]. Parks and Granger demonstrated that reperfusion following ischemia causes significantly greater intestinal mucosal injury than ischemia alone, suggesting that hypoxia primarily induces mucosal lesions during ischemia, while ROS and RNS contribute to additional damage during reperfusion [23,24]. The Chiu score analysis, which is used to assess the degree of intestinal damage, demonstrated that levosimendan pretreatment significantly mitigates mucosal I/R injury, indicating its protective role during both ischemia and reperfusion phases. Goblet cells are vital for intestinal barrier integrity, producing mucus that protects the small intestine from bacterial translocation and inflammation, especially after I/R injury [25]. Ischemia disrupts the mucus barrier, while increased goblet cell secretion and compound exocytosis rapidly counteract this, releasing stored mucus to protect intestinal crypts from bacterial invasion [26,27,28]. Alcian blue staining showed that levosimendan pretreatment increased goblet cell density, thus preserving the secretory function of the small intestine after I/R injury. These results are consistent with earlier studies reporting the protective effects of levosimendan on oxidative stress markers and histological damage during I/R injury [15,16].

The intestinal I/R injury model involving 30 min of ischemia and followed by 90 min of reperfusion is widely used and considered as a suitable approximation of human clinical conditions. Although complete reperfusion in humans typically takes 6–8 h, the accelerated metabolism in rats (approximately 6.4 times higher) makes a 90 min reperfusion period a relevant reflection of urgent clinical scenarios [29]. During intestinal I/R injury, a significant increase in the percentage of apoptotic cells has been observed, marked by elevated levels of cleaved CC3 and reduced expression of the anti-apoptotic protein B-cell lymphoma 2 (Bcl-2) in affected tissues [30]. Numerous studies have confirmed that CC3, a key pro-apoptotic factor, is activated following ischemia and initiates apoptosis, leading to cell death [31,32]. Consistent with these findings, immunohistochemical analysis revealed the highest intensity of CC3 immunoreactivity in the I/R group compared to controls. Importantly, pretreatment with levosimendan reduced CC3 levels, indicating a protective, anti-apoptotic effect during I/R injury. In the rat model of caecal ligation peritonitis, levosimendan treatment significantly reduced CC3 protein expression in affected tissues, leading to the assumption that a single early dose of levosimendan could be a promising therapeutic strategy to prevent organ dysfunction related to I/R injury and sepsis [33,34].

Multiple studies using intestinal, hepatic, and cardiac I/R models have demonstrated that pretreatment with levosimendan significantly reduces the apoptotic index, as evidenced by a decreased number of TUNEL-positive cells [35,36,37]. The findings of this study are consistent with previous research, demonstrating that levosimendan pretreatment significantly reduced the number of predominantly TUNEL-positive epithelial cells in intestinal tissue.

Nrf2 is a key transcription factor that maintains mucosal balance by limiting excessive ROS production and protecting against inflammation and mucosal damage through its antioxidant effects [38]. It supports cell survival and proliferation by regulating redox homeostasis, drug metabolism, and DNA repair [39]. Nrf2 controls enzymatic antioxidants like SOD, CAT, GSH, and HO-1, crucial for redox balance and cellular homeostasis, while reducing inducible nitric oxide synthase (iNOS) activation and protein kinase C activity to lower ROS and increase GSH levels, thus mitigating oxidative stress [22,40,41]. Recent studies indicated that Nrf2/HO-1 system activation might become an appropriate strategy to mitigate oxidative stress and inflammation to protect the organs from I/R injury via anti-inflammatory and anti-apoptotic effects [42,43,44]. The up-regulation of Nrf2 and HO-1 in the levosimendan pretreated group confirmed its protective effect against intestinal I/R injury. This is further supported by the interplay between Nrf2 and NF-κB, where Nrf2 inhibits NF-κB signalling and decreases pro-inflammatory cytokine expression, thereby reducing inflammation and apoptosis [45,46,47]. These findings are consistent with previous studies demonstrating the beneficial effects of levosimendan, which markedly enhances Nrf2 signalling in cerebral [21] as well as in renal, lung [33], heart [36], and liver I/R injuries [37].

The role of levosimendan in the treatment of acute heart failure is well known. As a non-catecholamine inotrope that does not increase cardiomyocyte cyclic AMP and oxygen consumption, it has become an excellent option for the treatment of cardiogenic shock [48]. Opening of potassium ATP channels of the inner side of mitochondria has been associated with cardioprotection, reduction in infarct size, and attenuation of I/R injury in animal model studies, as well in clinical studies. Levosimendan causes an increase in cardiac output and decrease in pulmonary capillary pressure, which is not accompanied by an increase in myocardial energy consumption [49,50]. Some studies concluded that levosimendan decreased the lipid peroxidation and apoptosis after I/R injury and during severe heart failure [51]. Our results are similar to previous study that showed protective effects of levosimendan on cardiomyocytes [36,52]. This was also confirmed in clinical settings, in which levosimendan improved outcomes after coronary artery bypass [53].

There are limited literal data about the effects of levosimendan on lung injury [54,55]. Some studies have shown that levosimendan administration caused a reduction in the apoptosis of lung tissue cells [54,55,56]. Our results are consistent with these reports.

Several animal studies have shown that levosimendan, in addition to improving heart function, dilates renal arteries and improves kidney blood flow. This effect leads to a reduction in kidney tissue lesions detected by histological and immunohistochemical analyses [57,58]. In our study the reno-protective effect of levosimendan was also confirmed.

## 4. Materials and Methods

### 4.1. Ethical Principles

All procedures were conducted with the approval of the Ethical Committee of the Medical Faculty of Banja Luka, in accordance with guidelines for working with experimental animals and ensuring animal welfare (Approval ID: 18/1. 331-3/23, dated 6 June 2023). Animal treatment was carried out in accordance with the Law of protection and welfare of animals of the Republic of Srpska (Official Gazette of the Republic of Srpska, No 111/08) and the directive of European parliament for conducting experiments on animals (210/63/EU), guidelines of Animal Research: Reporting of in vivo experiments (ARRIVE).

### 4.2. Experimental Animals and Protocols

**Figure 13:**
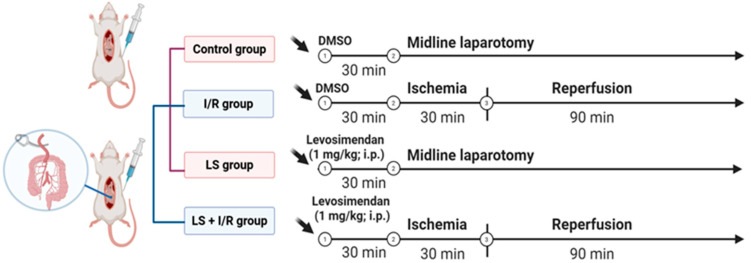
Illustration of the experimental study design. The schematic shows different experimental groups and timing of interventions (drug administration, midline laparotomy, ischemia, and reperfusion). Figure created with BioRender.com.

**Figure 14:**
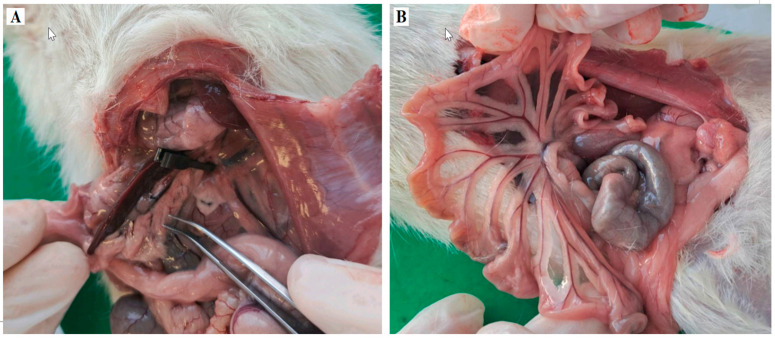
Representative photographs showing (**A**). SMA was exposed by carefully detaching from the surrounding tissue. The isolated SMA was occluded with an atraumatic artery microclamp (bulldog) at its branching from the aorta. (**B**) Ischemia of the intestine was verified by observation of dark discoloration of the intestinal loops (mostly ileum), loss of pulsation in mesenteric vessels, and visible intestinal atony (after 30 min of ischemia). Free peritoneum was protected by using a gauze soaked with warm 0.9% saline to minimise evaporative heat and fluid loss.

Male Wistar albino rats, weighing 280–320 g (8–12 weeks old), were kept under controlled laboratory conditions, at 21 ± 2 °C room temperature, 55 ± 5% humidity, and a 12 h light–dark cycle, with access to food and water “ad libitum”. Twenty-four animals were randomly divided into four groups. All animals underwent a midline laparotomy incision under general anaesthesia induced intraperitoneally with a combination of 90 mg/kg ketamine and 10 mg/kg xylazine. The sham group was used as a control (Control group, N = 6). The I/R group was used as the model disease for AMI, with clamping of the SMA for 30 min, followed by 90 min of reperfusion (I/R group, N = 6). The LS group received levosimendan (1 mg/kg. i.p.) and underwent the same sham procedures (LS group, N = 6). The LS + I/R group received levosimendan (1 mg/kg, i.p.) 30 min before the onset of I/R injury (LS + I/R group, N = 6). Rats from the Control and LS groups received DMSO as a solvent for levosimendan (Figure 13 and Figure 14). At the end of the experiment, all animals were sacrificed under deep anaesthesia by exsanguination performed through puncture of the thoracic artery, and blood and organ tissue samples were collected for further analysis.

### 4.3. Oxidative Stress Markers

Oxidative stress levels were assessed in plasma, erythrocyte lysate, BALF, and terminal ileum tissue homogenate. Plasma prooxidative markers, including H_2_O_2_, NO_2_^−^, and O_2_^−^, were quantified using the methods of Pick and Keisari [59], the Green method [60], and Nitro Blue Tetrazolium (NBT) reduction method [61], respectively. Lipid peroxidation was evaluated by measuring TBARS using 1% thiobarbituric acid (TBA) and 0.05 M sodium hydroxide (NaOH), with readings taken at 530 nm [62]. Antioxidant levels in erythrocyte lysate, including catalase CAT, SOD, and GSH, were measured spectrophotometrically according to Beutler’s methods [63,64,65].

### 4.4. Histopathological Examination and Morphometric Analysis

After 48 h of fixation in 4% formaldehyde, tissue samples were processed using a Leica TP 1020 tissue processor and embedded in paraffin blocks. Sections were cut to a thickness of 4 µm with a Rotatory 3003 pfm microtome and stained using routine haematoxylin and eosin staining, as well as with an Alcian Blue Stain kit (pH 2.5, mucin stain, Abcam, Cambridge, United Kingdom, CB2 OAX). The samples were analysed under a Leica DM 6000 binocular microscope equipped with a Leica DFC310FX camera. For morphometric analysis, the LAS V4.12 software was used at ×10 and ×20 magnification, depending on the type of tissue and organ, on 10 visual fields for each sample. Tissue damage score was determined semi-quantitatively as previously published [66,67,68,69]. Numerical areal density was determined for tissue slides stained with Alcian Blue. This density reflects the number of goblet cells in the terminal part of the small intestine relative to the entire tissue area. The visual field area was first calculated in square millimetres; then, the numerical areal density (NA) was calculated as the quotient of the number of structures or cells (N) to the area of the visual field (A), with NA = N/A. The resulting values are expressed as percentages.

### 4.5. Immunohistochemical Analysis

The activation of a specific apoptotic pathway was evaluated using immunohistochemical staining with primary antibodies NF-κB, CC3, Nrf2, and HO-1. Inflammatory markers were assessed by immunohistochemical analysis using primary antibodies to CD68 to confirm the presence of an immune cell infiltrate and ongoing immune response, as well as primary antibodies to IL-6 to identify a cytokine-mediated inflammation. Antibodies were applied to the samples and incubated overnight at 4 °C in a humid chamber. Detection was achieved using secondary antibodies conjugated with horseradish peroxidase (HRP) and a polyvalent detection system (UltraVision Detection System HRP Polymer & DAB Plus Chromogen, Thermo Fisher Scientific, 47777 Warm Springs Blvd. Fremont, CA, USA). Antibody binding sites became visible after staining with the chromogen 3,3′-diaminobenzidine tetrahydrochloride (DAB), and the samples were subsequently stained using the haematoxylin and eosin method.

Apoptosis was also analysed by the TUNEL method using a commercial TUNEL detection kit (TUNEL In Situ Kit, Elabscience, Wuhan, China; catalogue number: E-CK-A331) according to the manufacturer’s instructions. Briefly, paraffin sections were deparaffinised, rehydrated, and treated with proteinase K for permeabilisation. After washing, the tissue was incubated with a reaction solution containing TdT enzyme and labelled nucleotides at 37 °C for 60 min. Streptavidin-HRP conjugate was then added, and the signal was visualised using DAB substrate, with apoptotic cells shown as brown-stained nuclei. Sections were counterstained with haematoxylin, dehydrated, and mounted for light microscopic analysis. The apoptotic index (AI), representing the percentage of apoptotic cells, was calculated using the following formula:

AI (%) = (Number of TUNEL-positive cells × 100)/Total number of cells

All samples were examined under a Leica DM2500 optical microscope and captured with an MC170HD camera at 400× magnification. Microphotographs were archived in TIFF format. The immune response was analysed using Fiji software (Version 2.14.0/1.54f, National Institutes of Health, Bethesda, MD, USA), focusing on the number of DAB-positive cells and the mean optical density in the positive intestinal tissue. Results are presented as mean optical density ± standard deviation for 10 fields of view per rat, and the average optical density was compared between groups.

### 4.6. Statistical Analysis

Statistical analysis was performed with IBM-SPSS Statistic version 20.0 software (SPSS, Inc., Chicago, IL, USA), while GraphPad Prism 6.0 software was used for graphical representation. ANOVA test is used to compare the means of parametric characteristics and Kruskal–Wallis is used to compare the nonparametric characteristics between the groups. Tukey and Bonferroni tests are used for “post hoc” analysis. Results are presented as mean ± standard error, and the level of significance was set at *p* ˂ 0.05.

## 5. Conclusions

Levosimendan pretreatment exerts protective effects in intestinal I/R injury in rats through antioxidative, anti-inflammatory, and anti-apoptotic mechanisms. In our study, levosimendan administration during I/R significantly attenuated oxidative stress, as evidenced by reduced levels of TBARS, H_2_O_2_, NO_2_^−^, and O_2_^−^ and with increased activities of CAT and GSH. Histological analysis revealed a lower intestinal injury score and restoration of goblet cells, alongside reduced multi-organ tissue damage. Levosimendan downregulated pro-apoptotic markers NF-κB and CC3, alongside upregulation of the Nrf2 and HO-1 signalling molecules. These findings suggest that the protective effects of levosimendan are closely linked to signalling mechanisms responsible for suppression of pro-oxidative, pro-inflammatory, and pro-apoptotic responses, emphasizing its therapeutic potential in clinical settings.

### Limitation of the Study

The findings of this study suggest that levosimendan can mitigate mesenteric I/R injury and subsequent multi-organ damage by reducing oxidative stress, inflammation, and apoptosis. Nevertheless, the exact molecular mechanisms underlying these protective effects remain unclear, as no specific cellular target has yet been identified to account for its antioxidative, anti-inflammatory, and anti-apoptotic actions.

It is also important to note that these benefits were observed only under pretreatment conditions. This raises questions about their clinical relevance, since therapeutic administration after injury is of greater importance than prevention in the context of ischemia-reperfusion.

Consequently, future studies are needed to identify specific cellular targets underlying the molecular mechanisms of levosimendan’s mode of action and to evaluate its potential clinical efficacy when administered as a treatment.

## Data Availability

The authors confirm that the data supporting the findings of this study are available within the article.

## Abbreviations

AI	Apoptotic index
AMI	Acute mesenteric ischemia
ATP	Adenosine triphosphate
BALF	Bronchoalveolar lavage fluid
Bcl-2	B cell lymphoma-2
CAT	Catalase
CC3	Cleaved caspase 3
DAB	3,3′-diaminobenzidine tetrahydrochloride
DMSO	Dimethyl sulfoxide
GSH	Reduced glutathione
H_2_O_2_	Hydrogen peroxide
HO-1	Hem Oxygenase 1
HRP	Horseradish peroxidase
I/R	Ischemia/reperfusion
IL-1	Interleukin-1
Il-6	Interleukin-6
iNOS	Inducible nitric oxide synthase
LS	Levosimendan
MODS	Multiple Organ Dysfunction Syndrome
NaOH	Sodium hydroxide
NF-kB	Nuclear Factor kappa light-chain enhancer of activated B cells
NO_2_	Nitrogen dioxide
Nrf2	Nuclear factor erythroid 2- related factor
O_2_^−^	Super oxide anjon radical
OH	Hydroxyl radical
ONOO^−^	Peroxynitrite
RNS	Reactive nitrogen species
ROS	Reactive oxygen species
ROS/RNS	Reactive oxygen/nitrogen species
